# Effects of aripiprazole on prolactin levels and differences in effectiveness in patients with schizophrenia: a *post-hoc* analysis of the real-world data of a multicenter study

**DOI:** 10.3389/fpsyt.2024.1383173

**Published:** 2024-08-29

**Authors:** Qian Li, Yun-Ai Su, Xuemei Liao, Maosheng Fang, Jianliang Gao, Jia Xu, Mingjun Duan, Haiying Yu, Yang Yang, Zhiyu Chen, Jintong Liu, Shaoxiao Yan, Peifen Yao, Shuying Li, Changhong Wang, Bin Wu, Congpei Zhang, Tianmei Si

**Affiliations:** ^1^ Department of Psychopharmacology, Peking University Sixth Hospital, Peking University Institute of Mental Health, NHC Key Laboratory of Mental Health (Peking University), National Clinical Research Center for Mental Disorders (Peking University Sixth Hospital), Beijing, China; ^2^ Department of Psychiatry, Wuhan Mental Health Center, Wuhan, Hubei, China; ^3^ Department of Child and Adolescent Psychology, The Fourth People’s Hospital of Hefei, Hefei, Anhui, China; ^4^ Mental Health Center, The First Psychiatric Hospital of Harbin, Harbin, Heilongjiang, China; ^5^ Department of Science and Education, The Fourth People’s Hospital of Chengdu, Chengdu, Sichuan, China; ^6^ Department of Psychiatry, The Chinese People’s Liberation Army 904nd Hospital, Changzhou, Jiangsu, China; ^7^ Department of Psychiatry, Beijing Anding Hospital, Beijing, China; ^8^ Department of Psychiatry, Hangzhou Seventh People’s Hospital, Hangzhou, Zhejiang, China; ^9^ Department of Adolescent and Child Psychological Behavior, Shandong Mental Health Center, Jinan, Shandong, China; ^10^ Department of Integrated Chinese and Western Medicine, Beijing Huilongguan Hospital, Beijing, China; ^11^ Department of Psychiatry, Shanghai Mental Health Center, Shanghai, China; ^12^ Department of Psychiatry, The First Affiliated Hospital of Zhengzhou University, Zhengzhou, Henan, China; ^13^ Department of Psychiatry, The Second Affiliated Hospital of Xinxiang Medical University, Xinxiang, Henan, China; ^14^ Department of Psychiatry, Xi’an Mental Health Center, Xi’an, Shaanxi, China

**Keywords:** schizophrenia, aripiprazole, prolactin, efficacy, safety

## Abstract

**Objectives:**

To investigate the effect of aripiprazole on prolactin levels in patients with schizophrenia and analyze whether varying baseline prolactin levels affect the effectiveness and safety of aripiprazole, in a real-life diagnostic and therapeutic setting in a *post-hoc* analysis.

**Methods:**

In this *post-hoc* analysis, patients with schizophrenia in the acute phase were divided into an elevated-prolactin group and a normal-prolactin group. After 8 weeks of aripiprazole treatment, changes in the proportion of patients with an abnormal prolactin level were analyzed in both groups, and the efficacy and safety of aripiprazole were compared between the two groups.

**Results:**

The elevated-prolactin group had more women, a longer duration of disease, and lower Positive and Negative Syndrome Scale (PANSS) total and subscale scores at baseline compared with the normal-prolactin group (all P < 0.05), and there was no significant difference in the proportion of patients with prior use of antipsychotic medication between the two groups. Regardless of previous antipsychotic use, patients in both groups developed hyperprolactinemia (23/168 [13.7%] in those who had taken antipsychotics vs. 43/375 [11.4%] in those who had not). After 8 weeks of aripiprazole treatment, the proportion of patients with abnormal prolactin in the elevated-prolactin group significantly decreased with prolonged treatment (P < 0.001), and aripiprazole had no significant effect on the normal-prolactin group (P = 0.250). Normal-prolactin group showed better efficacy than the elevated-prolactin group, and the differences in efficacy between the two groups was observed from week 4 to the endpoint (all p<0.05). In total, 87.2% (68/78) patients experienced mild to moderate adverse events in the elevated-prolactin group, which was significantly more frequent compared with the normal-prolactin group 71.0% (365/514).

**Conclusions:**

In this real-world study, for patients with acute schizophrenia, aripiprazole was effective in lowering the proportion of patients with abnormal prolactin levels, while it had no significant effect on patients with normal baseline prolactin. After adjusting for factors such as sex, age, prior antipsychotic drugs use history and disease severity, effectiveness and safety of aripiprazole in patients with normal baseline prolactin was significantly better than that in patients with elevated baseline prolactin.

## 1 Introduction

Schizophrenia is a chronic, disabling disease. According to the Global Burden of Disease Study 2019, the global prevalence of schizophrenia is about 1% (1). Epidemiological studies in China show that the lifetime prevalence of schizophrenia is 0.6% (2). Schizophrenia accounts for about 50% of hospitalized psychiatric patients in China, which not only severely impairs patients’ own social functioning, but it also imposes a heavy burden on families and society (3, 4).

Antipsychotic medications are among the most effective treatments for patients with schizophrenia spectrum disorders (5–7). However, insufficient compliance is a problem with the use of antipsychotics, partly due to the various adverse reactions. According to a previous study, hyperprolactinemia (HPRL) or other endocrine-related adverse reactions are the common primary adverse drug reactions that result in a 31% decreased in patient adherence. Other common adverse reactions that lead to decreased patient compliance include agitation/extrapyramidal symptoms (EPS) (43%), metabolic adverse reactions (36%), and excessive sedation/cognitive impairment (30%) (8).

Hyperprolactinemia (HPRL) is a common adverse reaction of antipsychotics. The diagnostic criterion for HPRL is serum PRL level greater than 25 ng/mL (530 mIU/L) (9–11). Research has indicated that HPRL is present in 70% of patients with schizophrenia who are undergoing antipsychotic treatment (12, 13). The incidence of HPRL in China ranges from 42% to 90% in female psychiatric patients and from 18% to 72% in male psychiatric patients, and it is much higher than the incidence of HPRL in the general population (0.4%) (13).

A network meta-analysis by Leucht et al. provided a comprehensive comparison of the efficacy and tolerability of 15 antipsychotic drugs in treating schizophrenia. The results have indicated a higher risk of HPRL with the use of paliperidone, risperidone, and haloperidol, while aripiprazole had the lowest risk, even lower than placebo (14).

Aripiprazole is effective in treating HPRL caused by other antipsychotics. Consequently, several studies have investigated its use in modulating dopamine D_2_ receptors by switching or co-administering aripiprazole as a means of reducing prolactin levels in patients with HPRL (15, 16).

However, in real-life diagnostic settings, it remains uncertain whether aripiprazole consistently lowers prolactin levels in patients with various levels of baseline prolactin at the acute phase of schizophrenia. In addition, it is also unclear whether baseline prolactin levels affect the efficacy of aripiprazole. This *post-hoc* analysis examined the effect of aripiprazole on prolactin levels in patients with schizophrenia in a real-life diagnostic environment, and analyzed whether varying baseline prolactin levels influence the effectiveness and safety of aripiprazole.

## 2 Subjects and methods

### 2.1 Subjects

The participants in this study were selected from the *Pharmacologic Treatment of Acute Episode of Schizophrenia: A Real-World Study*. The research was led by Peking University Sixth Hospital, and 14 research institutions across China participated in the project. The study was approved by the Ethics Committee of Peking University Sixth Hospital (No. 2017-24) and was registered with Clinical Trials under the number NCT03289026. All of the subjects signed an informed consent form.

The inclusion criteria were as follows: (1) total Positive and Negative Syndrome Scale (PANSS) scores were ≥70 at baseline; (2) the individual item scored ≥4 for at least two of the following PANSS items: P1 Delusions, P2 Conceptual disorganization, P3 Hallucinatory behavior, and P6 Suspiciousness/Persecution; and (3) the PANSS-positive score was higher than the PANSS-negative score at baseline.

The exclusion criteria included: (1) concomitant unstable medical conditions or other mental diseases; (2) any evidence of suicide risk or history of violence; (3) previous allergic reaction to aripiprazole; (4) a history of neuroleptic malignant syndrome or severe extrapyramidal syndrome; and (5) women who were pregnant, planning a pregnancy, or breastfeeding; (6) refractory schizophrenia treated with electroconvulsive therapy within the past two months; and (7) participation in other clinical trials within 4 weeks before enrollment of this study.

### 2.2 Intervention

The eligible patients were given orally disintegrating aripiprazole tablets (Bosiqing^®^, Chengdu Kanghong Pharmaceutical Group Co., Ltd., Chengdu, Sichuan, China) for a period of 8 weeks, with four follow-up evaluations (at baseline and weeks 2, 4, and 8). The investigator was able to select a dosage between 10 and 30 mg/day, depending on the patient’s clinical needs. Taking any other antipsychotics, antidepressants, or mood stabilizers was strictly prohibited. Prior antipsychotic therapies had to be ceased within 2 weeks after the enrollment. Benzodiazepines, anticholinergics, and beta-blockers were permitted when clinically required, although benzodiazepines were not allowed to be used continuously for more than 7 days.

### 2.3 Data collection

The study protocol encompassed gathering demographic data and clinical characteristics of the enrolled patients; documenting aripiprazole usage; recording PANSS, Clinical Global Impression-Improvement (CGI-I), Clinical Global Impression-Severity of Illness (CGI-S), and Medication Satisfaction Questionnaire (MSQ) scores at baseline and at weeks 2, 4, and 8; and measuring PRL levels at baseline and at weeks 4 and 8; and measuring metabolism-related indexes levels (including body mass index [BMI], glucose, lipids) at baseline and at the end of week 8. Adverse events (AEs) were recorded during interviews and the Udvalg for Kliniske Undersogelser (UKU) Side Effects Rating Scale was used to evaluate the side effects induced by aripiprazole. Based on the *Consensus on the management of antipsychotic-induced hyperprolactinemia* (9–11), the enrolled patients were grouped according to their baseline prolactin levels into the elevated-PRL group (PRL ≥ 530 mIU/L) and the normal-PRL group (PRL < 530 mIU/L). Subjects in this study including patients who were already taking antipsychotics as well as those have not yet taken antipsychotics. We analyzed the proportion of patients with abnormal PRL levels in each group, and analyzed whether different baseline PRL levels affected the effectiveness and safety of aripiprazole.

### 2.4 Statistical methods

Effectiveness analyses were performed on the full analysis set (FAS). Safety analyses were based on the safety set (SS).

SAS 9.4 software (version 9.4, Cary, NC, USA) was used for statistical analysis. Continuous variables were presented as means (standard deviation [SD]) or medians (P25, P75), whereas categorical variables were presented as numbers (percentages). The response rate (defined as the rate of reduction from baseline in the PANSS total score>30%) and the improvement rate of CGI (defined as the proportion of patients with a CGI-I score of 1 and 2 after treatment) were estimated with the approximate normal method, providing their 95% confidence interval (CI) estimates. The mixed model for repeated measures (MMRM) analyses was used to compare the changes from baseline to endpoint in PANSS total score, PANSS score reduction rates, CGI-S, and MSQ, with sex, age, BMI, previous antipsychotic medication history, group, follow-up times, group×follow-up times and the baseline values as fixed effects, the patients as the random effect, and follow-up times as repeated measurement effect. The least square mean (LSM) with 95% CIs and standard error (SE) of the change were calculated. Based on the presence of elevated PRL levels (≥ 530 mIU/L) at baseline, the analyzed population was divided into two groups, namely, the elevated-PRL group and the normal-PRL group. Demographic characteristics and baseline clinical information of the two groups were compared using the Wilcoxon rank-sum test, Fisher’s exact test, or chi-square test, as appropriate. Logistic regression was performed to investigate the influence of baseline PRL levels on the effect of aripiprazole on metabolism-related indexes by calculating the odds ratios (ORs) with 95% CIs. Statistical significance was set at a two-sided P value of < 0.05.

## 3 Results

### 3.1 General information and clinical characteristics of the patients

Of 803 screened patients, a total of 703 patients satisfied the inclusion criteria.

The full analysis set (FAS) comprised 640 subjects (91.0%), and 537 subjects with documented baseline serum PRL levels were divided into the elevated-PRL group (N = 64) and the normal-PRL group (N = 473).

The safety set (SS) comprised 703 (100%) subjects, including 592 with documented baseline serum PRL level. Among these patients, 13.2% [78/592] presented with elevated PRL levels, while 86.8 [514/592] had normal PRL levels.

There was no significant difference in the proportion of patients with prior administration of antipsychotics between the elevated-PRL group and the normal-PRL group (38.2% vs. 29.8%, P > 0.05; **Table 1**). Meanwhile, regardless of whether the patients had previously taken antipsychotic medication, a certain proportion of patients had abnormal PRL levels at baseline (23/168 [13.7%] for those who had taken antipsychotics vs. 43/375 [11.4%] for those who had not).

**Table 1:** Baseline demographic and clinical characteristics of the elevated-prolactin group and the normal-prolactin group (FAS).

Statistics	Elevated-PRL group (N = 64)	Normal-RPL group (N = 473)	Total sample (N = 537)	P-value
**Sex, n (%)**	N=64	N=472	N=536	0.0004^a^
Male	15 (23.4)	221 (46.8)	236 (44.0)	
Female	49 (76.6)	251 (53.2)	300 (56.0)	
**Age (year)**	N=64	N=470	N=534	0.9414^b^
Mean (SD)	32.0 (10.44)	31.7 (9.97)	31.7 (10.02)	
**BMI (kg/m^2^),**	N=60	N=435	N=495	0.9390^b^
Mean (SD)	22.93 (4.220)	22.77 (3.556)	22.79 (3.638)	
**Duration of illness (year)**	N=57	N=432	N=489	0.0347^b^
Mean (SD)	2.31 (3.896)	2.16 (4.341)	2.18 (4.288)	
Median (P_25_, P_75_)	0.022 (0.003, 2.984)	0.003 (0.003, 2.313)	0.005 (0.002, 2.587)	
**Prior antipsychotics use history**	N=55	N=440	N=495	0.2024^a^
yes, n (%)	21 (38.2)	131 (29.8)	152 (30.7)	
**Baseline PANSS score, Mean (SD)**	N=64	N=473	N=537	
Total score	86.4 (15.98)	95.7 (19.43)	94.6 (19.27)	< 0.0001^b^
Positive score	25.1 (5.65)	28.0 (5.07)	27.6 (5.22)	< 0.0001^b^
Negative score	19.4 (6.57)	21.0 (6.48)	20.8 (6.51)	0.0201^b^
General psychopathology score	41.9 (8.35)	46.6 (11.42)	46.1 (11.19)	0.0017^b^
**Dosing regimen**	N=64	N=473	N=537	
Target dose (mg/day), **Mean (SD)**	28.1 (3.83)	28.9 (2.82)	28.8 (2.97)	0.5793^b^
**Target dose group, n (%)**	N=64	N=473	N=537	
≤ 15 mg/day	1 (1.6)	2 (0.4)	3 (0.6)	0.0748^c^
20 mg/day	8 (12.5)	25 (5.3)	33 (6.1)	
25 mg/day	5 (7.8)	49 (10.4)	54 (10.1)	
30 mg/day	50 (78.1)	397 (83.9)	447 (83.2)	
The initial dose (mg/day), **Mean (SD)**	11.5 (4.24)	10.9 (3.25)	11.0 (3.38)	0.5793^b^
**Time to target dose (days), Mean (SD)**	7.9 (4.79)	8.1 (4.67)	8.1 (4.69)	0.9928^b^

^a^Chi-square test; ^b^Wilcoxon rank-sum test; ^c^Fisher’s exact test.

SD, standard deviation; BMI, body mass index; PANSS, Positive and Negative Syndrome Scale; PRL, prolactin.

There were no significant differences in age and BMI between the elevated-PRL group and the normal-PRL group (both P > 0.05). Moreover, there was no significant difference between the two groups in terms of their dosing regimen, including the initial dose, target dose, or dosing speed (all P > 0.05). The elevated-PRL group had a higher proportion of women and longer duration of disease compared with the normal-PRL group. Furthermore, the PANSS total and subscale scores at baseline were lower in the elevated-PRL group (all P < 0.05), as indicated in **Table 1**.

### 3.2 Effect of aripiprazole on PRL levels in patients with schizophrenia

The proportion of patients with abnormal PRL in the elevated-PRL group significantly decreased with the duration of aripiprazole treatment (P < 0.001). However, aripiprazole did not exert a significant effect on patients with normal PRL levels (P = 0.250). The proportion of patients with abnormal prolactin in the elevated-prolactin group (N = 78) was 100%, 28.8%, and 25% at baseline, week 4, and week 8, respectively, and the number of missing cases was 0, 26, and 34, respectively; the proportion of patients with abnormal prolactin in the normal-prolactin group (N = 514) was 0%, 0.7%, and 0.8% at baseline, week 4, and week 8, respectively, and the number of missing cases was 0, 100, and 135, respectively.

### 3.3 Effectiveness analysis of aripiprazole in patients with different baseline PRL levels

All the patients, regardless of baseline PRL levels, showed significant reduction in PANSS total scores compared with baseline after 2 weeks of treatment, and the differences gradually increased with the duration of treatment (**Table 2**).

**Table 2:** Changes in key effectiveness indicators at different visiting points (FAS).

	Visit	Elevated-RPL group (N = 64)	95% CI	Normal-PRL group (N = 473)	95% CI	P-value
Change in PANSS score relative to baseline (LSM [SE])	Week 2	−29.55 (1.843)	−33.17, −25.94	−29.51 (0.660)	−30.80, −28.21	0.9817
	Week 4	−37.50 (1.913)	−41.25, −33.74	−43.09 (0.668)	−44.40, −41.78	0.0056
	Week 8	−45.95 (2.073)	−50.02, −41.89	−52.90 (0.679)	−54.23, −51.57	0.0014
PANSS score reduction rate (%) (LSM [SE])	Week 2	28.48 (1.730)	25.09, 31.87	30.40 (0.628)	29.17, 31.63	0.2931
	Week 4	37.03 (1.779)	33.54, 40.52	43.86 (0.633)	42.62, 45.11	0.0003
	Week 8	46.48 (1.892)	42.76, 50.19	53.40 (0.641)	52.15, 54.66	0.0005

LSM, SE, and P-values were derived from MMRM model.

LSM, Least square mean; SE, standard error; PRL, prolactin.

After 4 weeks of treatment, there was a significant difference in the reduction in the PANSS total score between the normal-PRL group and the elevated-PRL group (LSM [SE]: −43.09 [0.668] vs. −37.50 [1.913], P = 0.0056), and the differences persists until the end to the 8^th^ week (LSM [SE]: −52.90 [0.679] vs. −45.95 [2.073], P=0.0014) (**Table 2**).

At the end of 4^th^ week, the PANSS score reduction rate showed a significant difference between the normal-PRL group and the elevated-PRL group (LSM [SE]: 43.86% [0.633] vs 37.03% [1.779], P = 0.0003), and this significant difference also existed at the end of the 8^th^ week (LSM [SE]:53.40% [0.641] vs 46.48% [1.892], P=0.0005) (**Table 2**).

Meanwhile, the response rate in the normal-PRL group increased from 49.8% (226/454) at the 2^nd^ week to 96.6% (395/409) at the 8^th^ week. And that in the elevated-PRL group was 40.0% (24/60) at the 2^nd^ week, and it rose to 87.8% (36/41) at the 8^th^ week.

**Figure 1:**
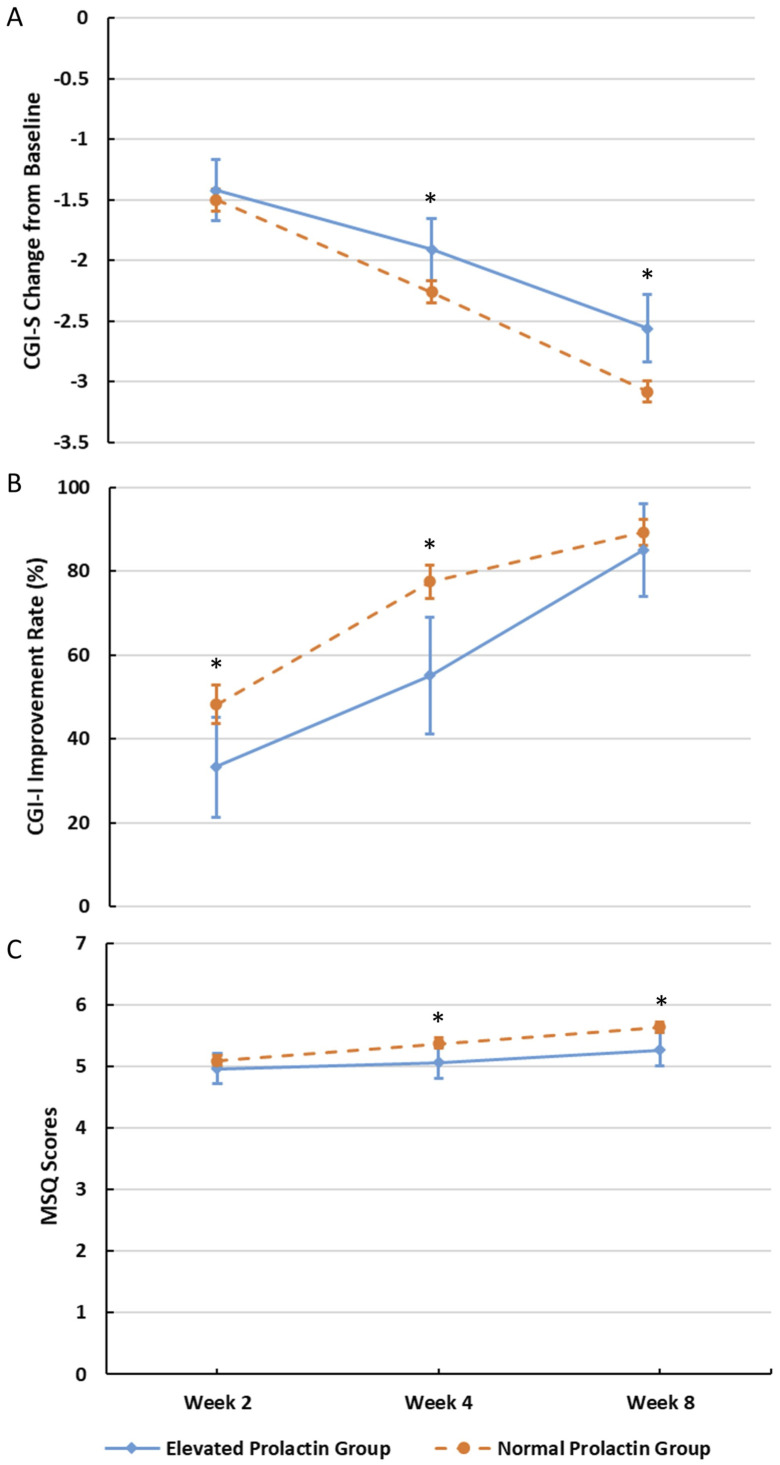
Changes in CGI-S, CGI-I, and MSQ at different visits. **(A)** Changes in CGI-S at different visits (*P<0.05). **(B)** Changes in CGI-I at different visits (*P<0.05). **(C)** Changes in MSQ at different visits (*P<0.05).

Further, after 2 weeks treatment, the changes of CGI-S score of patients with different baseline PRL levels were significantly different from baseline, and these differences continued to increase with the duration of treatment (**Figure 1A**).

At the end of 4^th^ week, there was significant difference in the CGI-S change from baseline between the normal-PRL group and the elevated-PRL group (LSM [SE]: −2.26 [0.046] vs LSM [SE]: −1.91 [0.133], P =0.0126), and this significant difference also existed at the end of the 8^th^ week (LSM [SE]: −3.08 [0.047] vs −2.56 [0.145], P=0.0006) (**Figure 1A**). After 4^th^ and 8^th^ weeks of treatment with aripiprazole, according to MSQ changes between the two groups, the treatment satisfaction of the normal-PRL group was better than that of the elevated-PRL group (P = 0.0235 and 0.0092; **Figure 1C**).

Additionally, the improvement rates of CGI-I increased over time for both groups. 216 (48.2%) and 333 (77.4%) patients in the normal-PRL group demonstrated improvement after 2 and 4 weeks of treatment, respectively. In contrast, only 20 (33.3%) and 27 (55.1%) patients in the elevated-PRL group showed improvement at the same time points (P for the intergroup differences = 0.0226 and 0.0025; **Figure 1B**).

### 3.4 Analysis of aripiprazole safety indicators in patients with various baseline PRL levels

Out of 592 patients, 57.9% (343/592) patients reported at least one AE associated with aripiprazole. Of those, 87.2% (68/78) were reported in the elevated-PRL group, while 71.0% (365/514) were reported in the normal-PRL group. The occurrence of AEs was significantly lower in the patients with normal PRL levels than in those with elevated PRL levels (P = 0.0027). According to the UKU scale, Akathisia was the most common adverse effects in both groups (37.2% vs 32.3%, the elevated-PRL group vs the normal-PRL group), the other AEs with an incidence of ≥10% included tremor (37.2% vs 27.2%), reduced sleep duration (14.1% vs 14.2%), tension or inner unrest (16.7% vs 12.6%), hyperkinesia (16.7% vs 10.5%), dystonia(16.7% vs 7.8%), and hypokinesia/akinesia(12.8% vs 5.4%), but were almost always mild to moderate.

As shown in **Table 3**, baseline PRL levels did not significantly influence the effect of aripiprazole on the proportion of patients with abnormal metabolism-related indexes.

**Table 3:** Abnormalities in metabolism-related indexes (SS).

Metabolism-related indexes	Statistics	Elevated-RPL group	Normal-PRL group
		(N = 78)	(N = 514)
BMI	Proportion of abnormal patients at baseline (%)	21/73 (28.8)	115/474 (24.3)
	Proportion of abnormal patients at week 8 (%)	13/44 (29.5)	102/389 (26.2)
	OR (95% CI)	1.255 (0.206, 7.660)	
Fasting blood glucose (mmol/L)	Proportion of abnormal patients at baseline (%)	11/78 (14.1)	37/512 (7.2)
	Proportion of abnormal patients at week 8 (%)	3/45 (6.7)	15/388 (3.9)
	OR (95% CI)	0.591 (0.073, 4.779)	
Total cholesterol(mmol/L)	Proportion of abnormal patients at baseline (%)	11/78 (14.1)	69/512 (13.5)
	Proportion of abnormal patients at week 8 (%)	5/45 (11.1)	53/389 (13.6)
	OR (95% CI)	1.202 (0.414, 3.493)	
Triglycerides(mmol/L)	Proportion of abnormal patients at baseline (%)	13/78 (16.7)	106/512 (20.7)
	Proportion of abnormal patients at week 8 (%)	9/45 (20.0)	106/389 (27.2)
	OR (95% CI)	0.707 (0.290, 1.720)	
High-density lipoprotein–cholesterol (HDL-C) (mmol/L)	Proportion of abnormal patients at baseline (%)	14/78 (17.9)	95/512 (18.6)
	Proportion of abnormal patients at week 8 (%)	8/44 (18.2)	48/389 (12.3)
	OR (95% CI)	1.033 (0.355, 3.005)	
Low-density lipoprotein–cholesterol (LDL-C)	Proportion of abnormal patients at baseline (%)	0/77 (0)	18/464 (3.9)
	Proportion of abnormal patients at week 8 (%)	0/43 (0)	3/343 (0.9)
	OR (95% CI)	–	

Abnormal fasting blood glucose: ≥ 6.1 mmol/L; Abnormal total cholesterol: ≥ 5.2 mmol/L; Abnormal triglycerides: ≥ 1.7 mmol/L; Abnormal HDL-C: < 1.0 mmol/L; Abnormal LDL-C: ≥ 4.1 mmol/L. ORs with 95% CIs were obtained through logistic regression models that were adjusted for sex, age, prior antipsychotic drugs use history, and baseline measurements.

BMI, Body mass index; FBG, Fasting blood glucose; TC Total cholesterol; TG,Triglycerides; HDL-C, High-density lipoprotein–cholesterol; LDL-C, Low-density lipoprotein–cholesterol; PRL, prolactin.

## 4 Discussion

Elevated prolactin levels or HPRL was common in schizophrenia patients receiving antipsychotics, which may raise the prolactin level by antagonizing the D_2_ receptor in the pituitary lactotroph cells. But in this study, we also found that a certain proportion (11.4%) of patients who did not use antipsychotic drugs had elevated prolactin levels, which far exceeds the percentage (0.4%) of HPRL reported in the general population (12). Riecher et al. (17) found that HPRL may be present in patients with schizophrenic psychoses independent of antipsychotic medication, consistent with the results obtained in this study. Previous research has highlighted variations in serum PRL secretion among individuals experiencing first-episode unmedicated schizophrenia, indicating that schizophrenia may entail anomalous regulation of PRL levels (17, 18). Yuan Xiuxia et al. (19) found a significant elevation in PRL levels among first-episode unmedicated patients with schizophrenia relative to healthy controls. Additionally, they proposed that the dysfunction of the hypothalamo-pituitary-gonadal axis may be implicated in the pathophysiological process of schizophrenia. The initial elevation in serum PRL levels can be seen as an organism’s response to stress, with an association between elevated serum PRL levels and the onset of schizophrenia. Segal et al. (20) reported elevated serum PRL levels among patients with first-episode and recurrent unmedicated hospitalized schizophrenia. No significant difference in serum PRL levels was found between first-episode and recurrent schizophrenia patients, indicating that elevated PRL levels may serve as a biomarker of schizophrenia.

Further, at baseline the patients with elevated PRL levels had less severe psychiatric symptoms than those with normal PRL levels, similar to the inverse relationship between basal PRL levels and the severity of positive symptoms found in patients with acute schizophrenia in a number of studies (20–22). Schwarz E and his colleagues attempted to identify any associations between PANSS scores and levels of serum molecules at baseline, they found that PRL levels showed a significant negative correlation with positive PANSS, indicating that patients with higher PRL levels had lower severity of symptoms at baseline (23).

However, the correlation between changes in serum PRL levels and psychiatric symptoms is still a subject of controversy (24). Yuan Xiuxia et al. (19) reported that serum PRL levels may indicate the severity of early clinical symptoms, while Yu Jianjin et al. (25) found that PRL levels were linked to psychopathological changes and sex differences in the association. Meanwhile, Wasnik et al. (16, 26) showed that there was no association between PRL levels and psychiatric symptoms in patients with schizophrenia. Additionally, She et al. (27) found no evidence of an association between PRL levels and psychiatric symptoms before and after treatment in schizophrenia patients. Further research may be required to fully investigate this relationship.

Aripiprazole is classified as a third-generation antipsychotic drug with unique pharmacological properties of dopamine D_2_ and serotonin 1A partial agonism and serotonin 2A antagonism (28), can ameliorate schizophrenia symptoms with fewer adverse effects (14). Our study revealed that treatment with aripiprazole did not result in a significant increase in serum PRL levels in patients with schizophrenia who had normal PRL levels at baseline. On the other hand, for patients with elevated PRL levels at baseline, aripiprazole successfully reduced serum PRL levels and decreased the proportion of patients with abnormal PRL levels among those with acute episode of schizophrenia, regardless of whether or not their elevated PRL levels had been caused by prior systemic treatment with antipsychotics, which is consistent with results of some previous studies. Lee et al. (29) demonstrated that after replacing amisulpride and risperidone with aripiprazole, PRL levels returned to normal and symptoms/signs of HPRL such as amenorrhea and galactorrhea reversed. Fujioi et al. (30) discovered that combined with aripiprazole to schizophrenia patients with HPRL resulted in reduced serum PRL levels and improved sexual function.

Our study found that aripiprazole was more effective in patients with normal serum PRL levels at baseline than in patients with elevated serum PRL levels at baseline, after adjusting for factors such as sex, age, prior antipsychotic drugs use history and disease severity. This may be related to the longer duration of disease in patients with elevated serum PRL levels at baseline. Altamura et al. (31) reported duration of disease had a negative effect on schizophrenic response (achievement of recovery/remission of symptoms, response to pharmacological treatment, reduction of suicidal risk, improvement of cognitive abilities and social functioning). Lo WH et al. (32) first reported that a shorter duration of disease positively affects schizophrenia outcome, defined as achieving remission of symptoms. And Loebel et al. (33) followed a group of first-episode schizophrenia patients for 3 years and they found that duration of disease before treatment was significantly associated with time to remission as well to the level of remission. However, it is unclear to figure the reason for the difference in efficacy between these two groups of schizophrenic patients treated with aripiprazole. This may be due to differences in disease duration or differences in PRL levels at baseline between the two groups, which remain to be further confirmed.

Our stratified analyses also found that aripiprazole had significantly lower occurrence of AEs in patients with normal PRL levels than those with abnormal PRL levels, which may be related to the shorter duration of disease in the group with normal PRL levels.

As for other metabolism-related indexes, we found that aripiprazole had no significant effect on fasting blood glucose, low-density lipoprotein, or high-density lipoprotein levels either in the elevated-PRL group or in the normal-PRL group. It slightly increased total cholesterol levels in the normal-PRL group and triglyceride levels in both groups of patients, but this was not clinically significant. And baseline PRL levels did not significantly influence the effect of aripiprazole on the other metabolism-related indexes.

Numerous long-term observational clinical and real-world studies (34, 35) have demonstrated the favorable effects of aripiprazole on patient satisfaction and adherence. These findings are consistent with the results of the present study. Given the high prevalence of nonadherence in severe mental illnesses such as schizophrenia, increased willingness to continue antipsychotic medication is an important step in maintaining mental stability (36).

In summary, the present study demonstrated that patients experiencing acute episode of schizophrenia in a real-world clinical environment may have elevated PRL levels, regardless of their previous antipsychotic medication. Treatment involving aripiprazole effectively reduces serum PRL levels in patients with elevated PRL levels and does not influence patients with normal PRL levels. By prolonging the treatment duration, aripiprazole has demonstrated significant effectiveness in decreasing the proportion of patients with abnormal PRL levels and increasing the proportion of patients with normal PRL levels. Hence, aripiprazole treatment proves to be both efficient and safe for patients undergoing acute episode of schizophrenia, despite varying baseline PRL levels. Notably, patients with normal PRL levels demonstrate better effectiveness with aripiprazole, compared with those with elevated PRL levels.

This study has some limitations due to the use of real-world data and as a *post-hoc* analysis. There are issues of uneven baseline, insufficient control of confounding variables, or impact on the interpretation of the results. The history of prior antipsychotic use (including type and dose of antipsychotics, etc.) and smoking history were not comprehensively documented for all of the patients, which may result in an inadequate examination of pertinent factors contributing to elevated PRL levels. Well-designed randomized controlled studies are still needed to confirm that aripiprazole is more effective in patients with normal PRL levels at baseline than in patients with elevated PRL levels at baseline, to explore possible reasons for this difference in efficacy, and to explore whether baseline PRL levels can be a predictor of efficacy for aripiprazole. Besides, as a *post-hoc* analysis, the relatively small number of patients in the elevated prolactin group compared to the normal prolactin group may limit the power to detect smaller effect sizes or differences. Nevertheless, the finding of a significant decrease in prolactin levels with aripiprazole in the elevated prolactin group, suggesting a robust effect of the study. Additionally, the 8-week follow-up in this study might not be sufficient to reflect the long-term outcome of patients with schizophrenia in real-world settings, and the long-term effects of aripiprazole on PRL require further investigation. So further well-designed studies with pre-specify sample size calculations and larger subgroup sizes, as well as long-term follow-up are also needed to validate and extend the findings of this study.

## Data Availability

The raw data supporting the conclusions of this article will be made available by the authors, without undue reservation.
